# Structure of the Human BK Ion Channel in Lipid Environment

**DOI:** 10.3390/membranes12080758

**Published:** 2022-07-31

**Authors:** Lige Tonggu, Liguo Wang

**Affiliations:** 1 Department of Biological Structure, School of Medicine, University of Washington, Seattle, WA 98195, USA; tglg@uw.edu; 2 Laboratory for BioMolecular Structure, Brookhaven National Laboratory, Upton, NY 11973, USA

**Keywords:** large-conductance voltage- and calcium-activated potassium (BK) channel, liposome, random spherically constrained (RSC), single-particle cryo-EM

## Abstract

Voltage-gated and ligand-modulated ion channels play critical roles in excitable cells. To understand the interplay among voltage sensing, ligand binding, and channel opening, the structures of ion channels in various functional states and in lipid membrane environments need to be determined. Here, the random spherically constrained (RSC) single-particle cryo-EM method was employed to study human large conductance voltage- and calcium-activated potassium (hBK or hSlo1) channels reconstituted into liposomes. The hBK structure was determined at 3.5 Å resolution in the absence of Ca^2+^. Instead of the common fourfold symmetry observed in ligand-modulated ion channels, a twofold symmetry was observed in hBK in liposomes. Compared with the structure of isolated hSlo1 Ca^2+^ sensing gating rings, two opposing subunits in hBK unfurled, resulting in a wider opening towards the transmembrane region of hBK. In the pore gate domain, two opposing subunits also moved downwards relative to the two other subunits.

## 1. Introduction

The large conductance voltage- and calcium-activated potassium (BK) channel belongs to the six-transmembrane-segment (6TM) ion channel family. It can be found in many cells, and functions as a feedback regulator of membrane potential and thereby Ca^2+^ influx [1,2,3]. Like other members of the 6TM ion-channel family, it has voltage-sensor domains (VSDs) that sense the transmembrane potential [4]. The BK channel is also a member of the Slo family whose α-subunits contain regulator-of-conductance-for- K^+^ (RCK) domains in the large intracellular C-terminal region. The crystal structures of the RCK domain of the *E. coli* K^+^ channel [5] and the gating ring of the *Methanobacterium thermoautotrophicum* K^+^ channel MthK [6,7,8] greatly aid in the understanding of the Ca^2+^-dependent regulation of Slo1 channels. These prokaryotic channel structures inspired a series of mutational studies demonstrating that the RCK1 and RCK2 domains in mouse Slo1 (mSlo1) account for the Ca^2+^-dependent activation of mSlo1 [9,10,11,12,13], and provided templates for the generation of homology models of the mSlo1 channel gating ring: the four RCK1 and four RCK2 domains from the four subunits in a functional Slo1 channel form a “gating ring”. There are two high-affinity Ca^2+^-binding sites in each subunit of the gating ring, with one in the RCK2 involving mD898, mD900, mQ892, and mD895, known as “Ca^2+^ bowl” [14,15], and the other in the RCK1 involving mD367, mE535, mR514 and several other residues [5,6,7,13,16]. In addition, there is a low-affinity Mg^2+^ binding site between the gating ring and the VSD involving mE374, mE399, and several other residues [10,12]. These findings were confirmed by the X-ray crystallographic structures of the isolated gating ring of the human Slo1 channel (hSlo1-GR) in the absence of Ca^2+^ [16] and that of the zebrafish Slo1 channel (zSlo1-GR) in the presence of Ca^2+^ [17]. Recently, the cryo-EM structures of the *Aplysia californica* Slo1 channel (aSlo1) were obtained with and without Ca^2+^, which further confirmed these findings [18,19]. All the gating rings in the structures consisting of RCK domains [5,6,7,15,16,17,18,19] had a fourfold symmetry along the central pore (C4). Although the two structures of aSlo1 were determined in both liganded and metal-free states [18,19], the structures of other states are useful. Functional studies show that the channel has at least four and probably many more conformational states, as the channel can be activated by Ca^2+^, voltage, or combinations of the two [3,4].

As shown by both structural and functional studies, the lipid membrane environment plays an essential role in the structural integrity and activity of membrane proteins [20,21,22,23,24,25]. Therefore, it is critical to restore the lipid membrane environment of membrane proteins. One method is to use lipid nanodiscs where the membrane protein resides in a small patch of a lipid bilayer encircled by an amphipathic scaffolding protein [26] or polymers [27]. The nanodisc method was employed to study the structures of ion channels [28,29,30,31,32,33,34,35,36,37,38,39], transporters [40,41,42], and other membrane proteins [43,44]. An alternative method is to reconstitute membrane proteins into liposomes. The liposome method was developed and employed to study the structures of large conductance voltage- and calcium-activated potassium (BK) channels [45,46], a voltage-gated potassium channel Kv1.2 [47] as well as the AcrB multidrug efflux pump of *E. coli* [48]. Although both methods restore the lipid environment of membrane proteins, there is a major difference: the liposome method mimics the cell and can potentially provide an asymmetric environment (i.e., inside and outside conditions can be varied independently), whereas there is only one environment surrounding the membrane proteins in the nanodisc method. This can be critically important for understanding membrane proteins that are controlled or modulated by asymmetric environment (e.g., applying transmembrane potential for voltage-gated ion channels or voltage-sensitive proteins, applying pressures to mechanosensitive channels).

Here, we employed the liposome method to study the structure of human BK (hBK) channels in the absence of Ca^2+^. By optimizing cryo-EM sample preparation, employing the direct electron detector and correcting the drift between frames with MotionCor2 [49], and utilizing random spherically constrained (RSC) single-particle cryo-EM, the resolution was improved from 17 to 3.5 Å [46], allowing for side-chain assignment for the gating ring and the pore gate domain. Compared with the commonly observed fourfold symmetry in all known gating ring structures, a broken symmetry was observed in the metal-free hBK channels reconstituted into liposomes. Within the tetramer, two opposing subunits in the Ca^2+^ sensing gating ring rotate around the center of mass of each subunit, which results in the movement of the assembly interfaces, flexible interfaces, and Ca^2+^ binding sites.

## 2. Materials and Methods

### 2.1. Protein Expression and Purification 

hBK (KCNMA1) proteins with FLAG-tagged on N terminus were stably expressed from HEK293 cells. Cell pellets from ~200 dishes were resuspended in Resuspend Buffer (50 mM TriHCl pH 7.4, 200 mM KCl, 5 mM EDTA, and protease inhibitor cocktail P8340 (Sigma-Aldrich, St. Louis, MO, USA) and homogenized with 100 strokes in a Dounce grinder (DWK Life Sciences, Millville, NJ, USA). The mixture was centrifuged at 38,400× *g* for an hour at 4 °C, and the pellets were then solubilized with Extraction Buffer (50 mM TriHCl pH 7.4, 200 mM KCl, 5 mM EDTA, 16 mM n-Dodecyl-beta-Maltoside (DDM), and protease inhibitor cocktail) for 2 h at 4 °C with gentle mixing. The mixture was centrifuged at 38,400× *g* for half an hour 4 °C, and the supernatant was incubated with Anti-FLAG M2 Affinity Gel (Sigma-Aldrich) for 1 h at 4 °C with gentle mixing. Resins were washed with Wash Buffer A (20 mM HEPES pH 7.4, 150 mM KCl, 5 mM EDTA, 8 mM DDM, and protease inhibitor cocktail) and Wash Buffer B (20 mM HEPES pH 7.4, 150 mM KCl, 5 mM EDTA, 4 mM n-Decyl β-Maltoside (DM), and protease inhibitor cocktail), and bound protein was eluted with Elution Buffer (20 mM HEPES pH 7.4, 150 mM KCl, 2 mM EDTA, 4 mM DM, 0.5 mg/mL FLAG peptides, and protease inhibitor). Eluted protein was concentrated to 1 mL using Amicon Ultra-4 Centrifugal Filter100 kD MWCO (Millipore, Burlington, MA, USA), and further purified with Superose 6 Increase size-exclusion chromatography (Cytiva, Marlborough, MA, USA) using Running Buffer (20 mM HEPES pH 7.4, 150 mM KCl, 2 mM EDTA, 4 mM DM). The peak fractions were concentrated for the reconstitution step (Figure S1A) and evaluated using SDS-PAGE (Figure S1B).

### 2.2. Reconstitution of hBK Channels into Liposomes

The DM-solubilized hBK proteins were reconstituted into 1-palmitoyl-2-oleoyl-glycero-3-phosphocholine (POPC) liposomes with a detergent removed by gel filtration [50]. The 125-kDa hBK monomer is detected by SDS-PAGE before and after reconstitution (Figure S1B). Briefly, purified hBK was mixed with POPC stock (10 mM POPC (Avanti Polar Lipids, Alabaster, AL, USA) and 30 mM DM) at a lipid-to-protein ratio (LRP) 1 K:1 for 2 h at 4 °C with gentle mixing. The mixture was then loaded to a Sephadex G50 (Cytiva) column with Running Buffer (20 mM HEPES pH 7.4, 150 mM KCl, 2 mM EDTA) at 0.25 mL/min at room temperature. The proteoliposome fractions were collected, swelled, and concentrated to 1 mg/mL (protein) for further vitrification. The reconstitution yielded an average protein content at one to two hBK tetramers per 20-nm liposome. As the reconstitution of hBK proteins into liposomes was expected to restore the lipid environment of membrane proteins, the function of the reconstituted channels was confirmed using a flux assay with fluorescent dye 9-amino-6-chloro-2-methoxyacridine (ACMA) (Sigma-Aldrich), which was employed to assess the functions of reconstituted ion channels [51,52,53,54] (Figure S1D,E).

### 2.3. CryoEM Grid Prepartion, Data Collection, and Data Processing

**Figure 1:**
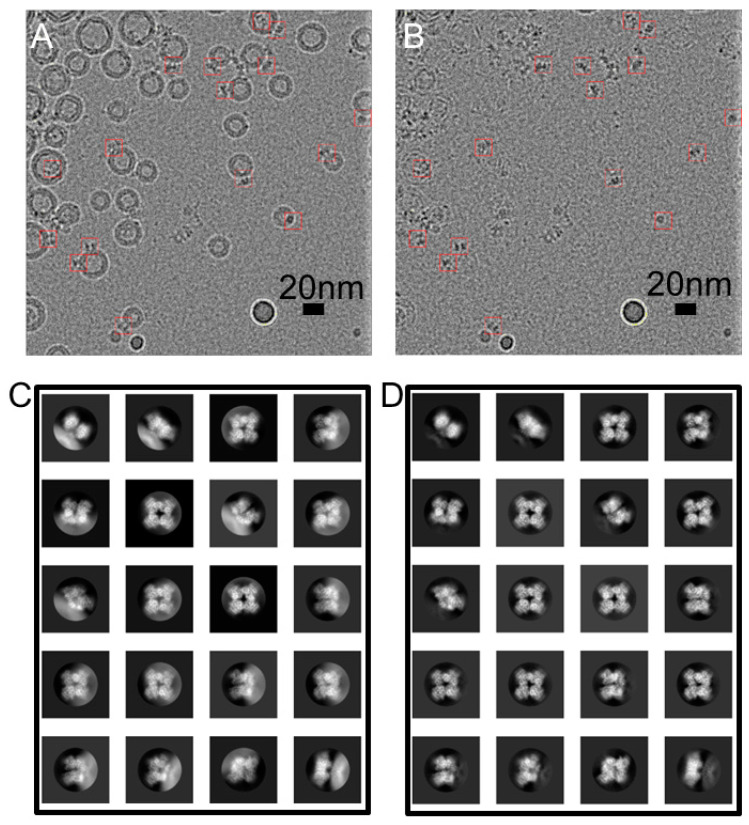
Subtraction of modeled liposome images from an hBK proteoliposome micrograph. (**A**) A representative cryo-EM image of hBK proteoliposomes at −3.8 μm defocus; (**B**) liposomes are subtracted from (**A**). hBK particles are marked with red boxes (15 nm); (**C**,**D**) 2D class averages of BK particles (**C**) before and (**D**) after liposome subtraction. Box size was 27 nm, and the circular mask was 17 nm in diameter.

The single-particle reconstruction of unstained cryo-EM specimens typically requires the acquisition of hundreds of thousands of particles. Because of the low yield of hBK expressed in HEK293 cells, and thus the low concentration of proteoliposomes, acquiring such a large number of protein particles in liposomes is challenging. Previously, a 2D streptavidin crystal was used as an affinity surface to tether the biotinylated-lipid-doped proteoliposomes [46]. However, empty liposomes bind preferentially to the crystal, further reducing the number of visible protein particles. We developed a method for liposome grid preparation [55] to increase particle density. Briefly, we employed a long-incubation method (a 5–10 min incubation on holey carbon grids (Quantifoil Micro Tools GmbH, Großlöbichau, German) followed by another application of sample before freezing) with a MarkIV Vitrobot (Thermo Fisher Scientific, Waltham, MA, USA). On average, there were 26 liposomes in each cryo-EM micrograph, which covered 0.16 μm^2^ of the specimen (Figure 1A). 

Cryo-EM data were recorded on a Titan Krios microscope (Thermo Fisher Scientific) operated at 300 kV, equipped with a GIF-quantum energy filter (Gatan, Pleasanton, CA, USA) at 20 eV slit width and a K2 Summit direct detector (Gatan). LEGINON [56] was used for automated data collection. In total, 3628 videos were collected at a nominal magnification of 130,000× in super-resolution mode, resulting in a pixel size of 0.525 Å. The total exposure time was 8.6 s (0.2 s/frame), leading to a total accumulated dose of 60 electrons per Å^2^. Dose-fractionated super-resolution image stacks were motion-corrected with MotionCorr2 [49]. Each frame in the image stack was divided into 5 × 5 patches for anisotropic image motion correction, and dose weighting was carried out to calculate the motion-corrected image. CTFFIND4 [57] was applied to estimate the contrast transfer function parameters for each motion-corrected image.

Approximately 4800 particles from 350 micrographs were interactively selected and classified using RELION [58]. Ten representative classes were selected as the templates for automated particle picking using RELION. Unfortunately, variability in liposome size precludes the merging of their images; instead, we fitted and subtracted a POPC liposome model [45] of the membrane contribution to each image (Figure 1B). Protein particles were picked from the resulting liposome-subtracted micrographs and were classified using RELION [58]. The automatically picked particles were screened using homemade MATLAB (MathWorks, Natick, MA, USA) programs to exclude particles more than 80 A away from any liposome, which resulted in 482,935 particles from 2786 micrographs. Some structural details are visible in the 2D classes (Figure 1C,D). The presence of a lipid membrane was confirmed in the 2D classes of protein particles extracted from original cryo-EM micrographs using the orientation information determined with liposome-subtracted protein particle images (Figure 1C,D).

**Figure 2:**
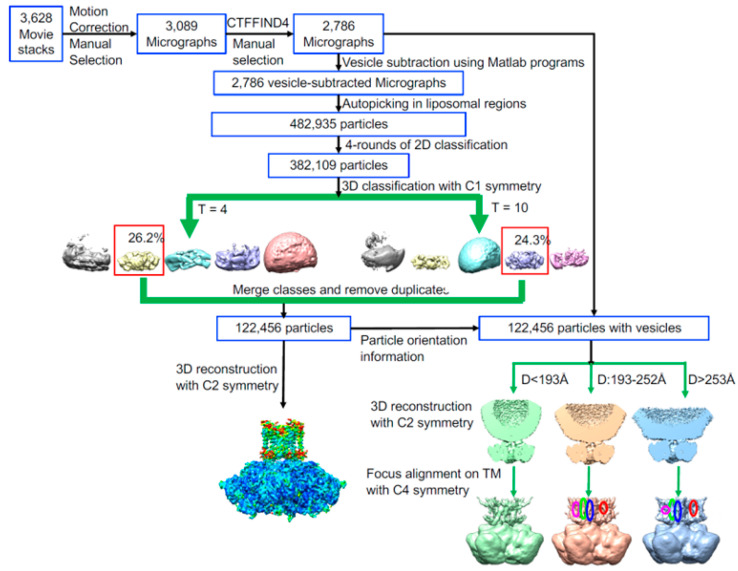
Flowchart for data processing.

After rounds of 2D and 3D classification in RELION, 122,456 particles were selected for the final 3D reconstruction (Figure 2). The resolution was estimated using the Fourier shell correlation (FSC) between independently refined half-maps with a criterion value of 0.143 [59]. The reconstruction of the BK structure carried out without symmetry (C1), and with twofold (C2) and fourfold symmetry (C4) applied yielded maps at resolutions of 3.79, 3.48, and 3.45 Å, respectively (Figure S3A).

As the 3D reconstruction without symmetry revealed C2 symmetry, we built an atomic model with the 3.48 Å map with C2 applied. PDB model 5TJI [19] was docked into a cryo-EM density map using UCSF Chimera [60]. The atomic model was then built and refined using Phenix [61].

## 3. Results

### 3.1. Overall Structure of hBK in Liposomes

**Figure 3:**
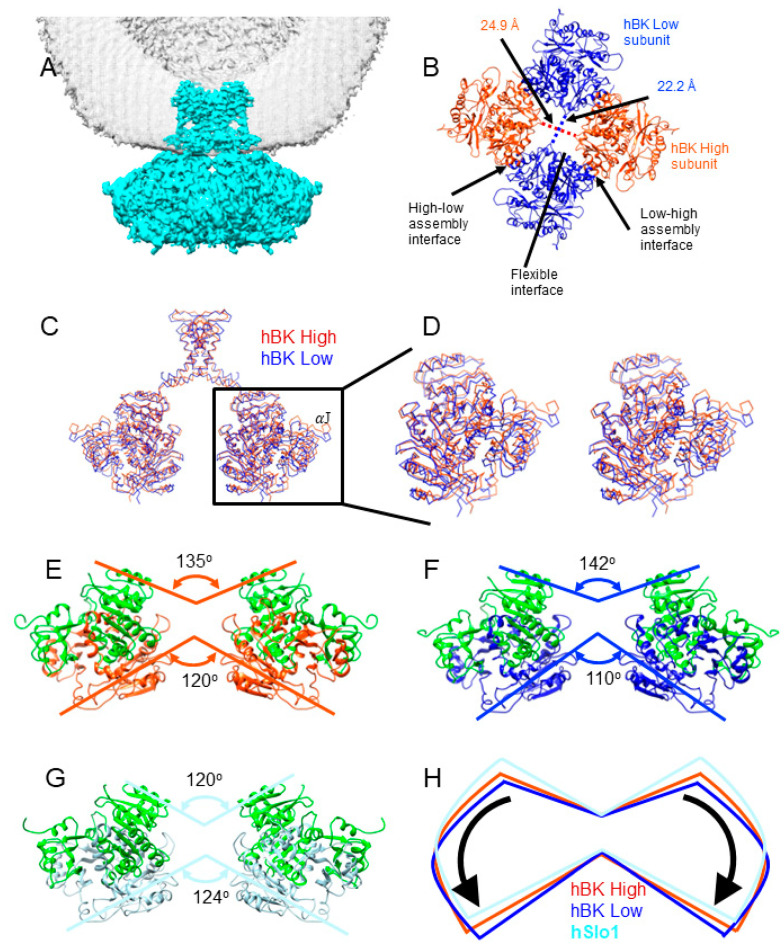
The structure of hBK in liposomes. (**A**) hBK cryo-EM density map. Lipid membrane is shown in gray mesh. For clarity, only half of the lipid membrane is shown; (**B**) view of the gating ring in hBK from the extracellular side. The diagonal distances between the C_α_ atoms of Val 785 are indicated; (**C**) superposition of hBK high (orange red) and low (blue) subunits. The shoulder helix J is indicated; (**D**) stereo view of the superimposed hBK high and low subunits; (**E**–**G**) side views of hBK high € (red), hBK low (**F**) (dark blue), and hSlo1-GR (**G**) (light blue, PDB: 3NAF). To distinguish RCK1 and RCK2 domains, the RCK1 domains in all three models were colored green. The RCK2 domains in hBK high subunits are aligned to those in hSlo1-GR; (**H**) cartoon to show the relative rotation among hBK high and low, and hSlo1-GR.

A twofold symmetry was observed (Figure 3) in the reconstruction without any symmetry applied, and agreed well with the reconstruction with C2 applied. In the structure, the central opening of the gating ring defined as the distance between C_α_ atoms of V785 in opposing subunits differs by 2.7 Å along the two diagonals (Figure 3B). The shoulder helix J moved by about 5 Å from two opposing subunits, termed as hBK high, to the two other opposing subunits, termed as hBK low (Figure 3C). This movement is comparable to the movement of the gating ring between the metal-free and liganded aSlo1 structures [18,19]: the metal-free gating ring moved ~5 Å away from the TM region. Due to the rotational flexibility of the transmembrane (TM) domain as observed in metal-free aSlo1 [19], the TM helices are proportionally displaced to the distance to the ion-conducting pore (e.g., 6 Å at helix S1 and 9 Å at helix S0). As a result, only helices S5 and S6, and the pore domain were determined together with the gating ring using the C2 symmetry. 

**Figure 4:**
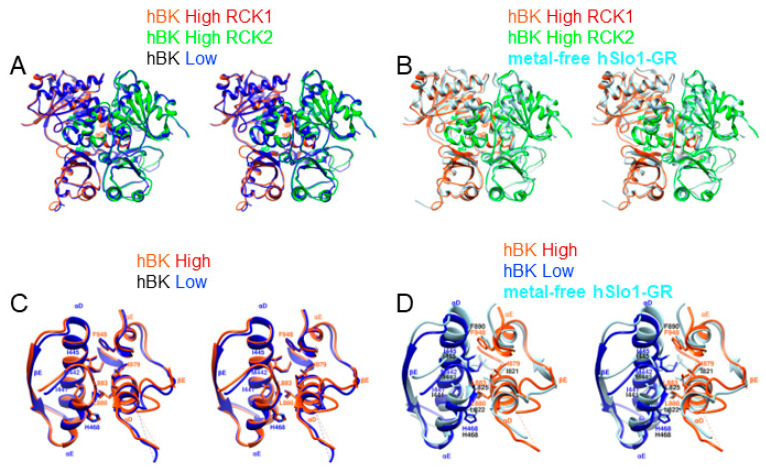
Stereo views of assembly and flexible interfaces in hBK. (**A**) Flexible interfaces in hBK. hBK high (orange red) and hBK low (dark blue) subunits are superimposed. To show the flexible interface, RCK2 in hBK high subunit is colored green; (**B**) Same as (**A**) except the replacement of hBK low with hSlo1-GR (light blue, PDB: 3NAF) subunit; (**C**) Assembly interface in hBK as defined in Figure 3B. hBK high (orange red) and low (blue) subunits are superimposed. The interacting residues are annotated; (**D**) Same as (**C**) except the replacement of hBK low with hSlo1-GR (light blue, PDB: 3NAF) subunit. The αD and αE helices in RCK2 of hSlo1-GR were aligned to those of hBK.

Compared with the structure of the isolated hSlo1 gating ring (hSlo1-GR) [16], a significant conformational change was observed (Figure 3E–H, Video S2). In general, the RCK1 domains in hBK opened like the petals of a flower, but the extent of the opening was different in hBK high and low subunits. The two RCK1 domains in hBK high subunits opened 15 degrees more than that in hSlo1-GR at the top (close to the membrane), while the RCK1 domains opened 22 degrees more than that in hSlo1-GR (Figure 3E–G). As RCK1 domains opened, the RCK2 domains also underwent conformational changes. The RCK2 domains at the bottom (away from the membrane) in hBK high subunits close dby 4 degrees more than the corresponding domains of hSlo1-GR, while the RCK2 domains of hBK closed 14 degrees more than that of hSlo1-GR (Figure 3E–G). The extent of the opening of RCK1 at the top differed from the extent of the closing of RCK2 at the bottom, even in the same subunit. This difference was not due to the relative movement between RCK1 and RCK2 domains in each subunit. As shown in Figure 4A, the flexible interfaces formed by the RCK1 and RCK2 domains in the hBK high and low subunits were the same after alignment. The difference of the opening at the top and closing at the bottom in the hBK high and low subunits was due to the rotation and tilting of each subunit, as shown in Video S2. As a result, the high–low and low–high assembly interfaces that formed between neighboring subunits remained the same. The interacting residues (I441, M442, I445, H468, L880, L883, I879 and F948) aligned well with each other (Figure 4C, Video S3). In short, the flexible and assembly interfaces in hBK remained the same in the hBK high and low subunits. As hBK channels were reconstituted into liposomes while isolated hSlo1 gating ring formed crystals, some changes were observed in both the flexible and assembly interfaces (Figure 4B,D). At the flexible interfaces, the positions of the helix-crossover domain (αF-turn-αG) that interlocked the two RCK domains changed from hSlo1-GR to hBK, but the folding was maintained. At the assembly interfaces, the αD and αE helices in hBK low subunits shifted down (away from the membrane) with respect to those in hSlo1-GR (Figure 4D). The changes were not significant; thus, the interaction between neighboring subunits should not change.

### 3.2. An Intermediate State of the BK Channel

**Figure 5:**
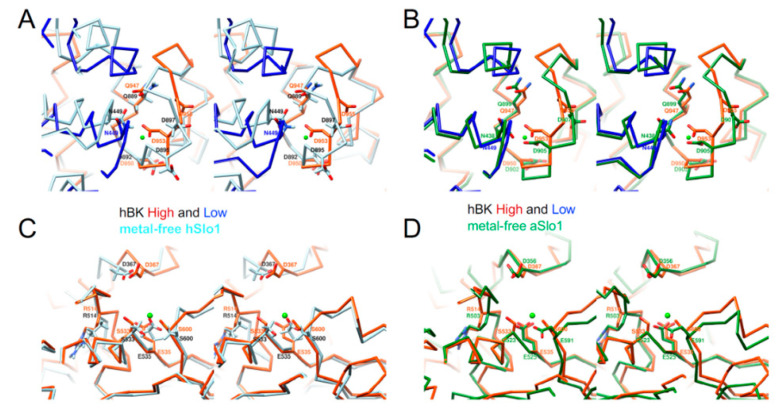
Stereo views of Ca^2+^-binding sites in hBK. (**A**) Ca^2+^ bowl in metal-free hBK (orange-red and blue) and metal-free hSlo1-GR (light blue, PDB: 3NAF); (**B**) same as (**A**), except metal-free hSlo1-GR was replaced with metal-free aSlo1 (green, PDB: 5TJI); (**C**) RCK1 Ca^2+^-binding site in metal-free hBK and metal-free hSlo1-GR (light blue, PDB: 3NAF); (**D**) same as (**C**), except metal-free hSlo1-GR was replaced with metal-free aSlo1 (green, PDB: 5TJI). Ca^2+^ coordinating residues were labeled, and the position of Ca^2+^ ion in liganded aSlo1 is represented by a green sphere.

There exist two high-affinity Ca^2+^-binding sites in hBK: the Ca^2+^ bowl and the RCK1 Ca^2+^-binding site. At the Ca^2+^ bowl, residues D953, D955, and N449 (D905, D907, and N438 in aSlo1), and the backbone carbonyl of D950 and Q947 (D902 and Q899, and in aSlo1) were expected to provide the basis to coordinate a Ca^2+^ ion, as seen in liganded aSlo1 (Figure 5B). In the absence of Ca^2+^, the Ca^2+^ bowl in hBK (same conformation in both hBK high and low subunits) differed significantly from that in hSlo1-GR (Figure 5A): N449 and D953 moved towards the Ca^2+^ binding pocket, while D955 swung away from the Ca^2+^ binding pocket. Similar movements of those three residues (N438, D905, and D907) were observed in metal-free aSlo1 (Figure 5B). At the low-affinity Ca^2+^ binding site, residues D367, E535, S600, and S533 (D356, E525, and E591 in aSlo1) (S533 in hBK replaced G523 in aSlo1), and the backbone carbonyl of R503 (R503 and G523 in aSlo1) were expected to provide the coordination of a Ca^2+^ ion, as seen in aSlo1. In metal-free hBK, D367 moved closer to the Ca^2+^ binding pocket (Figure 5C) than D367 in hSlo1-GR. As seen at the Ca^2+^ bowl, a similar movement of D367 (D356 in aSlo1) was observed in metal-free aSlo1 (Figure 5D). In short, both Ca^2+^ binding sites in hBK differed from those in hSlo1-GR, but agreed with those in aSlo1.

### 3.3. Rotational Flexibility in the TM Region

**Figure 6:**
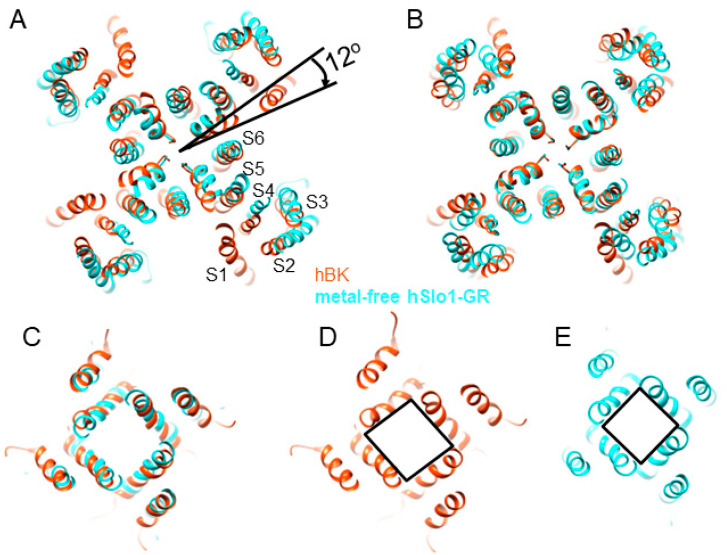
Comparison of the TM region in hBK in liposomes in the absence of Ca^2+^ and metal-free aSlo1 (PDB: 5TJI). (**A**) TM region in hBK (orange red) rotated by 12 degrees clockwise with respect to the main 3D class from the aSlo1 dataset (cyan); (**B**) aSlo1 rotated by 12 degrees to overlay with hBK. All views are from the extracellular side; (**C**) superposition of the intracellular helix bundle crossing region of hBK and aSlo1; (**D**,**E**) intracellular helix bundle crossing region of hBK and aSlo1, respectively.

In the analysis of metal-free aSlo1 cryo-EM data, alternative 3D maps were obtained that show an 8-degree variation in the rotation angle of the TM region relative to the gating ring [19]. We obtained only one 3D class, but presumably due to a similar rotational flexibility of the transmembrane (TM) domain, helices S0–S4 could not be well-resolved in the EM density map with a C2 symmetry. Imposing a C4 symmetry, however, produced a stronger signal (Figure 2 and Figures S2). Our hBK structure shows a total rotation of 12 degrees relative to the major 3D class from aSlo1 (Figure 6A). Helix S0 observed in metal-free aSlo1 was not visible in hBK, as it was the farthest from the symmetry axis. As shown in Figure 6A, VSD was more extended when compared with that in metal-free aSlo1. As metal-free aSlo1 was solvated in detergents, VSDs may adopt a more compact conformation. In this study, hBK was reconstituted into liposomes, and the lipid bilayer environment was restored. As the lipid membrane was fluidic, the protein had more flexibility; thus, the VSDs adopted a more extended conformation.

Upon rotating the aSlo1 TM region by 12 degrees, the pore domain of aSlo1 overlapped with that of hBK (Figure 6B). As this pore domain was closer to the central symmetry axis of the channel and experienced less blurring (Figure S2), the intensity in the pore domain was much stronger that that in the VSD regions. Thus, the pore domain was visible in the reconstruction with a C2 symmetry (Figure 3 and Figure 6C,D). The intracellular helix bundle crossing of hBK formed a parallelogram instead of a square in aSlo1 (Figure 6C–E). This was due to the movement of the RCK domains in the gating ring region (Figure 3C,D).

## 4. Discussion

Each BK channel α-subunit contains two RCK domains in its large intracellular C-terminal region. The X-ray crystallographic structure of metal-free hSlo1-GR revealed a gating ring formed by 4 RCK1 and 4 RCK2 domains [16], similar to those observed in the *E. coli* K^+^ [5], the MthK [6,7,8] and aSlo1 [18,19] channels. When hBK channels were reconstituted into liposomes in the absence of Ca^2+^, the fourfold symmetry was broken, and a twofold symmetry was observed. In each hBK subunit, the flexible interface was the same as that in hSlo1-GR (Figure 3B). To accommodate the broken symmetry, the assembly interfaces between neighboring subunits, twisted as shown in Figure 3D and providing the interactions between critical residues were not changed. Regarding Ca^2+^-binding sites, larger conformation changes were observed between hBK and hSlo1-GR. At the Ca^2+^ bowl, residues N449 and D953 moved towards the Ca^2+^ binding pocket, and D955 swung away from the Ca^2+^ binding pocket. At the RCK1 Ca^2+^ binding site, D367 moved towards the Ca^2+^ binding pocket. The origin of the difference may have been due to crystal constraints as both Ca^2+^ binding sites in hBK were similar to those in aSlo1, determined using cryo-EM. As the gating ring in aSlo1 also had a fourfold symmetry, a similar opening of RCK1 and closing of RCK2 were observed from the metal-free aSlo1 to hBK. The hBK high subunits had a similar distance to the membrane as that in aSlo1, while the hBK low subunits were farther away from the membrane. The origin of the downward movement of the hBK low subunits or the twofold symmetry instead of the fourfold symmetry might lie in the restoration of the lipid membrane environment. 

On the basis of metal-free and liganded aSlo1 structures, Hite et al. proposed a model of Slo1 gating by Ca^2+^ and voltage [19]. In the presence of Ca^2+^ under positive transmembrane potential (e.g., 120–240 mV), the gating ring adopts a compact conformation, similar to liganded aSlo1 in detergents [18]. VSDs are up, and the channel is open. In the absence of Ca^2+^, the gating ring adopts a loose conformation and is away from the membrane, as seen in ligand-free aSlo1 in detergents [19]. As the transmembrane potential drops to zero, two VSDs move downward (maybe not fully down as in the hyperpolarized membrane), thus pushing the two opposing subunits away from the membrane (hBK low subunits). This is consistent with the opening step that occurs cooperatively among the subunits [62,63] and comprises at least two transitions [64]. To confirm this hypothesis, the structure of hBK reconstituted in liposomes in the presence of Ca^2+^ is at least needed.

The proteoliposomes used for structure determination ranged from 15 to 70 nm in diameter, with the mean diameter at 20 nm (Figure S1C). The question, therefore, arises, whether the membrane curvature in liposomes of different sizes would affect the observed hBK structure, especially the C2 symmetry. To address this question, three hBK structures were reconstructed from small (15–19 nm), intermediate (19–25 nm), and large (25–70 nm) proteoliposomes (Figure 2 and Figure S2). The gating ring agreed well in the three reconstructions, and all showed the C2 symmetry. A parallel reconstruction with the liposome information-unsubtracted particle images illustrates the curved membrane (Figure S2). The reconstructions from hBK reconstituted in intermediate and large liposomes show similar VSD helices, in addition to the pore region, as that in the reconstructions from hBK reconstituted in all the liposomes. The pore region in the reconstruction from hBK reconstituted from small liposomes agrees well with other reconstructions, but the VSD was much noisier. Thus, the membrane curvature only affected the VSD region when the liposomes were smaller than 19 nm in diameter.

## 5. Conclusions

Full-length human large conductance voltage- and calcium-activated potassium (hBK or hSlo1) channels were reconstituted into liposomes and the random spherically constrained (RSC) single-particle cryo-EM method was employed to determine its structure. Instead of the common fourfold symmetry observed in ligand-modulated ion channels, a twofold symmetry was observed in hBK in the liposomes. Within the tetrameric channel, two opposing subunits in the Ca^2+^ sensing gating ring rotated around the center of each subunit, which resulted in the movement of the assembly interfaces, flexible interfaces, and Ca^2+^ binding sites. In the pore gate domain, two opposing subunits also moved downwards relative to the two other subunits. The new hBK structure presents a transition state where two VSDs moved downwards and pushed the two opposing subunits away from the membrane. This finding not only demonstrates the importance of the presence of a lipid membrane environment, but is also of great value to others working in this area.

## Data Availability

The coordinate of the atomic model reported in this paper was deposited in the Protein Data Bank (PDB) under accession code 6ND0. The cryo-EM map was deposited in the Electron Microscopy Data Bank (EMDB) under accession code EMD-0439. Any additional information required to reanalyze the data reported in this work paper is available from the lead contact upon request.

## Supplementary Materials

Supplementary materials can be downloaded at: https://www.mdpi.com/article/10.3390/membranes12080758/s1.
