# Supplementary material for: Structure of the Human BK Ion Channel in Lipid Environment
Source: Membranes (Basel). 2022 Jul 31;12(8):758. doi: 10.3390/membranes12080758 (PMC9414842; doi:10.3390/membranes12080758)
Supplement: Supplementary file 1 [file membranes-12-00758-s001.zip › membranes-1829524-supplementary.pdf]

SUPPLEMENTAL INFORMATION

SUPPLEMENTAL FIGURE LEGENDS

Figure S1. Purification, liposome reconstitution and functional flux assay.

(A) Purification of hBK by size-exclusion chromatography. The red box indicates peak fractions collected for liposome reconstitution; **(B)** SDS-PAGE gel image of hBK after purification step and after reconstitution step was stained by Coomassie Blue; **(C)** Histogram of hBK proteoliposomes radius, measured by using CryoEM micrographs and self-written script in MATLAB; **(D)** Cartoon illustrating the steps for Ion Flux Assay. Step1, potassium ions flux out to external solution due to electrochemical gradient. Step2, protons flux into the liposomes to counter the efflux of potassium ions via protonophore Carbonyl cyanide 3-chlorophenylhydrazone (CCCP). Step3. membrane permeable sensitive dye ACMA bind to protons and cannot diffuse out of liposomes anymore. ACMA fluorescence is quenched; **(E)** Representative ion flux assay data with initial Potassium gradient [K^{+}_{in}]/[K^{+}_{out}] set at 10 and 100.

Figure S2. Cryo-EM maps focused alignments.

(A) A zoom-in for Figure 2. 122,456 particles on micrographs before liposome subtractions were used to perform Focus Alignment in RELION. Based on the liposome size, the 122,456 particles were partitioned to 3 subsets evenly. The three subsets were then subject to RELION focus alignment on TM (with C4) and on Gating Ring (C2); (**B)** The 122,456 particles on micrographs before liposome subtractions subsets were subject to RELION focus alignment on TM (with C4). With different threshold, S1-S6 or S5&S6 can been seen.

Figure S3. Resolution of the cryo-EM Maps.

(A) The gold-standard Fourier Shell Correlation (FSC) curves for hBK in liposomes with C1, C2, C4 symmetry applied during the final reconstruction; **(B)** Local Resolution map for hBK in liposomes with C2 applied.

SUPPLEMENTAL MOVIES LEGENDS

Movie S1. The rotation of Cryo-EM map of hBK in liposomes with C2 applied.

Movie S2. Morphing between the hBK High (red) and Low subunits (dark blue) and hSlo1 (PDB 3NAF).

Movie S3. Morphing between the hBK High and Low then back to High. Red is RCK1 and Green is RCK2.

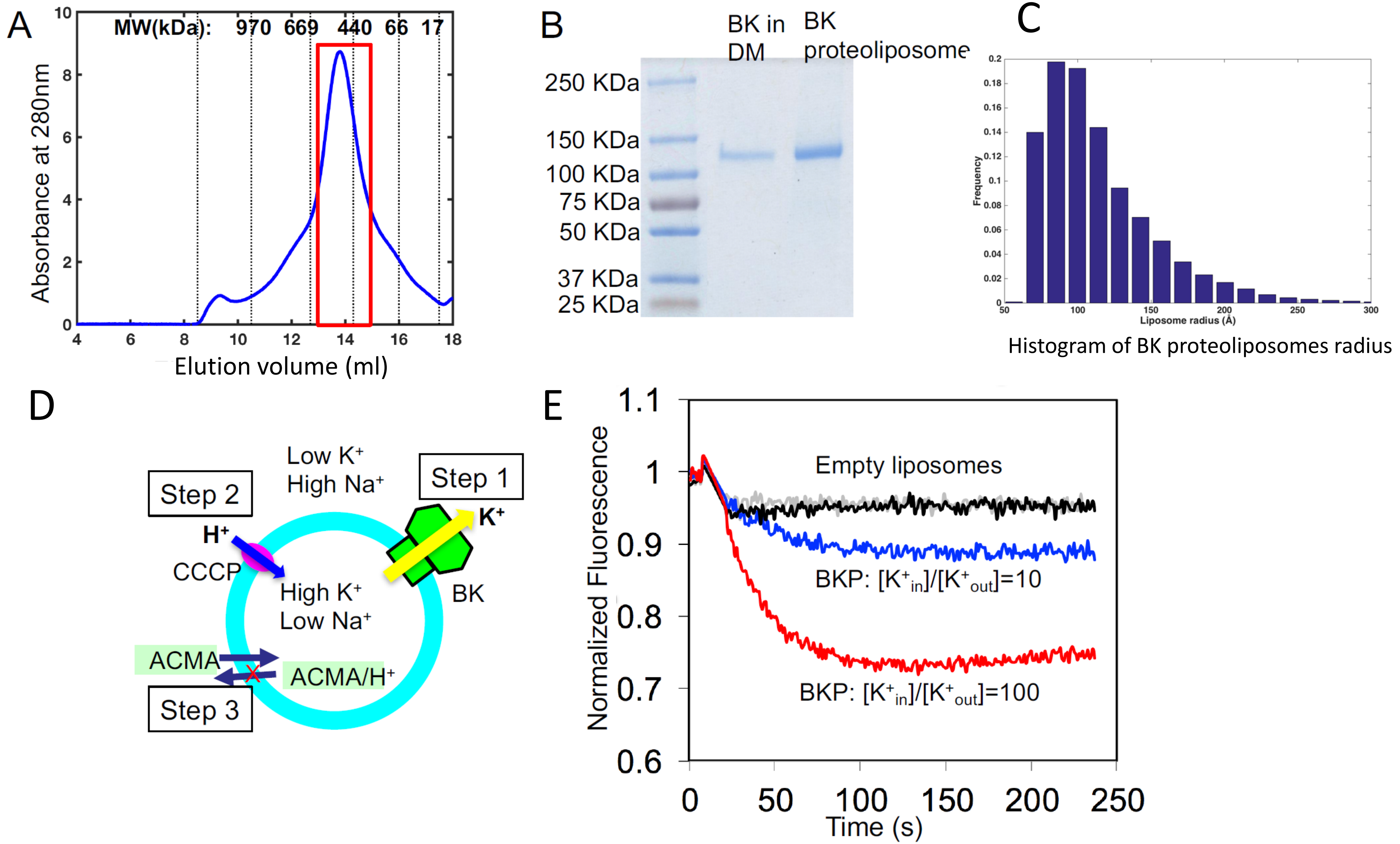
A
MW(kDa): 970 669 440 66 17
Absorbance at 280nm
Elution volume (ml)
B
BK in DM
BK proteoliposome
250 KDa
150 KDa
100 KDa
75 KDa
50 KDa
37 KDa
25 KDa
C
Frequency
Liposome radius (Å)
Histogram of BK proteoliposomes radius
D
Step 1
Step 2
Step 3
Low K+
High Na+
High K+
Low Na+
H+
CCCP
K+
BK
ACMA
ACMA/H+
E
Normalized Fluorescence
Time (s)
Empty liposomes
BKP: [K+in]/[K+out]=10
BKP: [K+in]/[K+out]=100

Figure S1

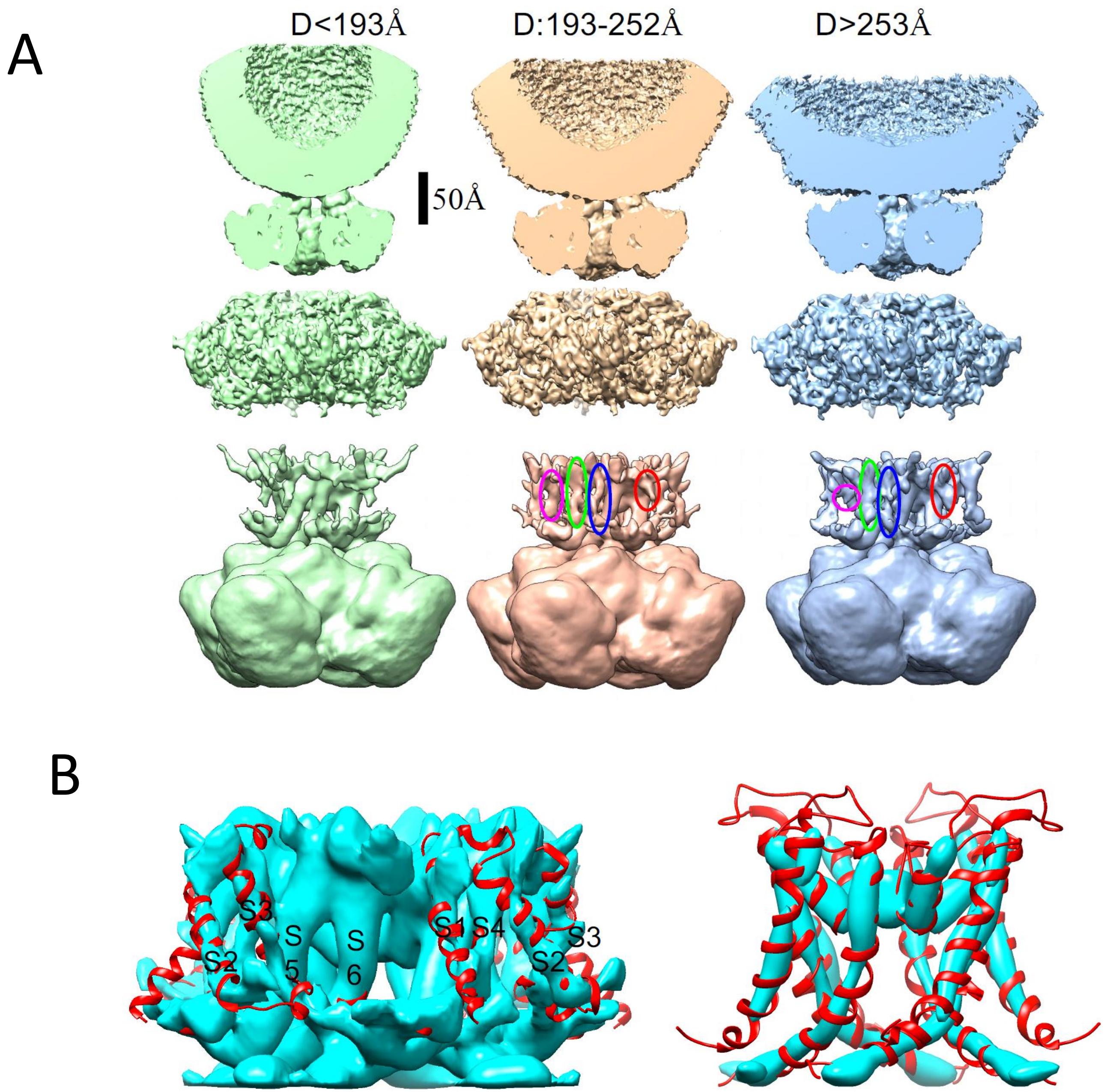

Figure S2

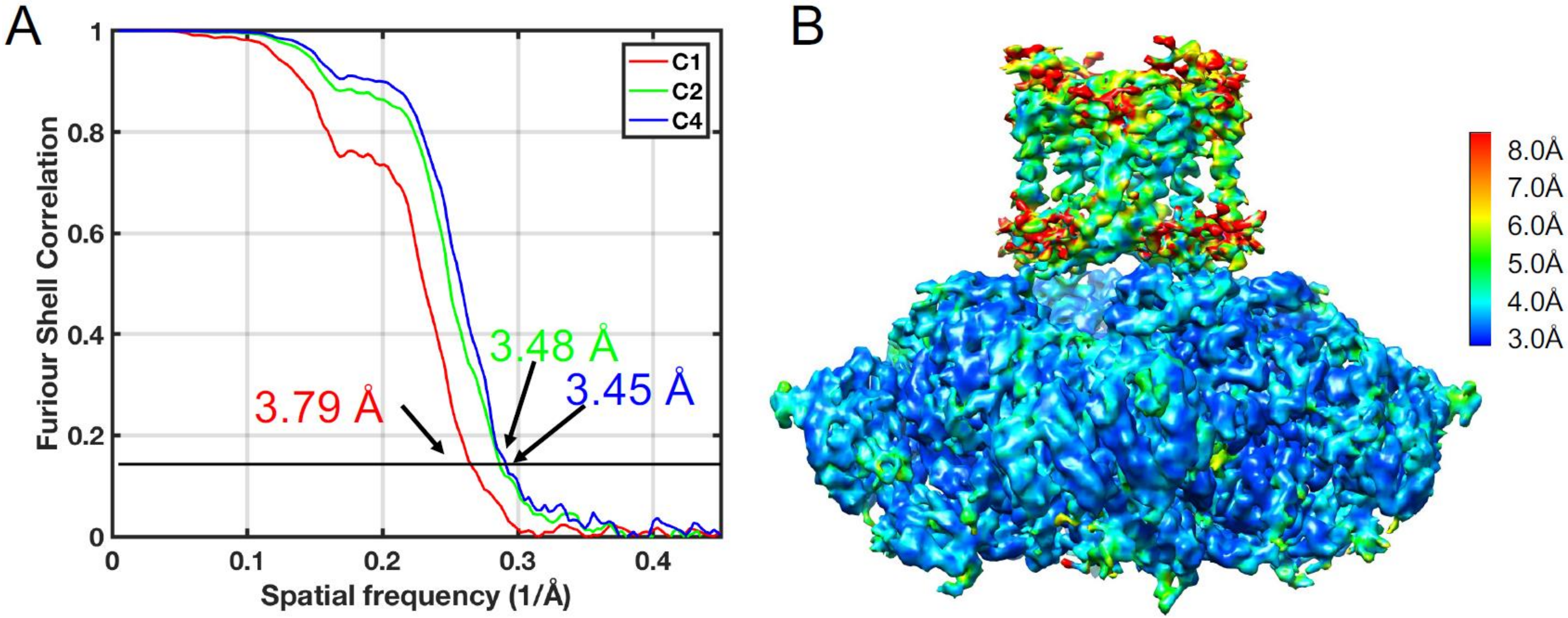
A
B
Furiour Shell Correlation
Spatial frequency (1/Å)
C1
C2
C4
3.79 Å
3.48 Å
3.45 Å
0
0.1
0.2
0.3
0.4
0.2
0.4
0.6
0.8
1
8.0Å
7.0Å
6.0Å
5.0Å
4.0Å
3.0Å

Figure S3
